# Cosuppression of NF-κB and AICDA Overcomes Acquired EGFR-TKI Resistance in Non-Small Cell Lung Cancer

**DOI:** 10.3390/cancers14122940

**Published:** 2022-06-14

**Authors:** Min-Kyung Yeo, Yoonjoo Kim, Da Hye Lee, Chaeuk Chung, Go Eun Bae

**Affiliations:** 1 Department of Pathology, School of Medicine, Chungnam National University, Daejeon 35015, Korea; mkyeo83@gmail.com; 2 Division of Pulmonology, Department of Internal Medicine School of Medicine, Chungnam National University, Daejeon 35015, Korea; k1206j@cnuh.co.kr (Y.K.); ziczi02@naver.com (D.H.L.)

**Keywords:** NF-κB, AICDA, EGFR-TKI, lung adenocarcinoma

## Abstract

**Simple Summary:**

Since the first discovery of EGFR-tyrosine kinase inhibitors (TKIs), they have become the gold standard treatment for EGFR-mutated non-small cell lung cancer. However, the inevitable acquisition of secondary TKI resistance after treatment with TKIs remains an unresolved issue. Here, we evaluated the expression of NF-κB, AICDA, Akt, IL-6, Jak2, and Stat3 by EGFR-TKI-resistant lung adenocarcinoma (LAC), and found that NF-κB and AICDA are major players in the acquired resistance of lung cancer to TKIs. Therefore, treatment with an EGFR-TKI plus cosuppression of NF-κB and AICDA may be a promising strategy to overcome EGFR-TKI resistance in LACs.

**Abstract:**

Background: Acquired resistance after EGFR-tyrosine kinase inhibitor (TKI) treatment is the rule rather than the exception. Overcoming resistance to EGFR-TKIs is essential if we are to develop better therapeutic strategies for lung cancer patients. Here, we examine the effector signaling pathways underlying TKI resistance and propose targets to overcome the resistance of lung adenocarcinoma (LAC) to TKI. Methods: We compared the expression of NF-κB, AICDA, Akt, IL-6, Jak2, and Stat3 by EGFR-TKI-resistant and EGFR-TKI-sensitive LAC cell lines, and by LAC patients treated with EGFR-TKIs; we then evaluated links between expression and treatment responses. We also examined the therapeutic effects of NF-κB and AICDA inhibition in EGFR-TKI-resistant LACs. Results: NF-κB and AICDA were more expressed by EGFR-TKI-resistant LACs than by EGFR-TKI-sensitive LACs. EGFR-TKIs induced a dose-dependent increase in the expression of NF-κB, AICDA, and IL-6. Inhibition of NF-κB suppressed the expression of AICDA, Akt, and IL-6 in EGFR-TKI-resistant and EGFR-TKI-sensitive LACs, whereas knockdown of AICDA suppressed the expression of NF-κB and Akt in both cell types. Treating EGFR-TKI-resistant LACs with an EGFR-TKI, alongside cosuppression of NF-κB and AICDA, had a significant therapeutic effect. Conclusion: Treatment with an EGFR-TKI plus cosuppression of NF-κB and AICDA may be a promising strategy to overcome EGFR-TKI resistance in LACs.

## 1. Introduction

After the first discovery of epidermal growth factor receptor (EGFR) oncogenic mutations in non-small cell lung cancer (NSCLC), EGFR-tyrosine kinase inhibitors (TKIs) became the standard treatment [1]. However, most NSCLC patients treated with EGFR-TKIs inevitably experience disease progression due to acquired drug resistance [2,3,4,5]. The most common mechanism of drug resistance is the acquisition of secondary site EGFR mutations such as T790M, which occurs after treatment with first- and second-generation TKIs, and C797S, which occurs after treatment with third-generation EGFR-TKIs [6,7,8,9]. The downstream pathways arising from secondary EGFR mutations are to reactivate major oncogenic intracellular signals, thereby promoting cancer progression. However, the mechanisms underlying the acquisition of additional mutations, and the related effector signal pathways, remain unclear.

Recent studies have shown that NF-κB activation in lung cancers treated with EGFR inhibitors promotes the survival of tumor cells and induces the development of drug resistance [10,11]. Previous studies suggest that EGFR-TKIs induce NFκB activation, which in turn induces TKI resistance through several pathways, including activation-induced cytidine deaminase (AICDA), interleukin-6 (IL-6)/JAK2/STAT3, and Akt [10,12,13,14]. Kadi and colleagues showed that the secondary EGFR T790M mutation is acquired through the upregulation of NF-κB signaling and AICDA enzymes [12]. They showed that activated NF-κB promotes the expression of AICDA, which induces the deamination of 5-methylcytosine to thymine at position 2369 and generates the EGFR T790M mutation in lung cancer cell lines [12]. In addition, IL-6 induces gefitinib resistance in NSCLC, mediated through the JAK2/STAT3 pathway via NF-κB signaling activated by EGFR-TKI [10,13]. Akt also regulates the transcriptional activity of the NF-κB pathway, and the EGFR-TKI resistance of EGFR-mutated NSCLC is attenuated by inhibiting Akt activity [14].

Herein, we hypothesize that EGFR-TKI induces activation of the NF-κB, Akt, IL-6, Jak2, Stat3, and AICDA signaling pathways, and that accumulation of these interactive signals results in resistance to TKIs. Therefore, we examine the relationship between NF-κB, Akt, IL6/JAK2/STAT3, and AICDA under EGFR-TKI treatment, and propose target markers for overcoming the EGFR-TKI resistance of NSCLC.

## 2. Materials and Methods

### 2.1. Cell Lines and Cell Viability Assay

The human lung adenocarcinoma (LAC) cell lines PC9 (a human LAC cell line harboring the EGFR-exon 19 deletion) and PC9/GR (a human LAC cell line harboring the EGFR T790M mutation, which is resistant to EGFR-TKI therapy) were cultured at 37 °C/5% in RPMI-1640 medium (WELGENE) containing 10% fetal bovine serum (FBS) (WELGENE). The EGFR-TKI gefitinib was from Tocris (Bristol, UK) (Iressa, 184475-35-2), and Bay11-7082 (D5556) was from Sigma. Cell viability was measured with gefitinib and Bay11-7082 using the CCK-8 assay kit (Dojindo Laboratories, Kumamoto, Japan) as previously described [15,16,17,18,19].

### 2.2. Western Blot Assay

Cells were harvested and suspended in protein lysis buffer (Translab, Daejeon, Korea). The protein concentration was measured using a Bio-Rad protein assay (Bio-Rad, cat.no.500-0006). Next, 30 μg protein (μg/mL) was separated on a 10% SDS-PAGE gel and transferred to a polyvinylidene difluoride (PVDF) membrane (Millipore, Burlington, MA, USA). The membrane was probed with the following antibodies: anti-β-actin (sc-47778, Santa Cruz Biotechnology, Dallas, TX, USA), anti-AICDA (ZA001, Invitrogen, Waltham, MA, USA), anti-p-NF-kB (p-p65) (#3033, Cell Signaling Technology, Danvers, MA, USA), anti-NF-kB (p65) (#8242, Cell Signaling Technology), anti-NF-kB (p105/50) (ab7549, Abcam, Cambridge, UK), anti-NF-kB (p100/52) (#4882, Cell Signaling Technology), anti-RelB (#10544, Cell Signaling Technology), anti-p-AKT (#4060, Cell Signaling Technology), anti-AKT (#9272, Cell Signaling Technology), anti-p-STAT3 (Tyr705, Cell Signaling Technology), anti-p-JAK2 (ab195055, Abcam), anti-JAK2 (#3230, Cell Signaling Technology), anti-p-EGFR (Try1045, #2237, Cell Signaling Technology), anti-p-EGFR (Tyr1068, #2236, Cell Signaling Technology), and anti-EGFR (sc-373746, Santa Cruz Biotechnology). Blots were developed using an enhanced chemiluminescence detection kit (Bio-Rad, Hercules, CA, USA).

### 2.3. Real-Time Reverse Transcription Polymerase Chain Reaction (RT-PCR)

Cells were collected, and total RNA was isolated, using TRIzol reagent (Invitrogen). Briefly, cDNA was synthesized from mRNA using Oligo(dT) primers. Quantitative PCR was carried out using a QuantiSpeed SYBR No-Ros kit (PhileKorea, Seoul, Korea) and the CFX Connect Real-Time PCR Detection System (Bio-Rad). All experiments were performed in triplicate. Relative expression of the indicated mRNAs was determined using the 2^−ΔΔCT^ method and normalization to β-actin levels. Expression of IL-6 mRNA was assessed by real-time reverse transcription polymerase chain reaction (RT-qPCR). Detailed information for the primer is listed in Supplementary Table S1.

### 2.4. Lentivirus Production, Infection, and Knockdown of AICDA Genes

Lentiviruses were prepared in HEK293T cells. PC9 and PC9GR cells were infected with the lentivirus to knockdown human AICDA genes. Expression-arrest pLKO lentiviral vectors encoding non-silencing control AICDA shRNA were provided by Sigma (TRCN0000412426, TRCN0000050344, and TRCN0000424739). Virus supernatant was added to cells (70–80% confluent) along with polybrene (with 2 ng/mL). Noninfected cells were eliminated by puromycin selection. The sequences of the shRNAs are as follows:

shAICDA#1, 5′-CCGGACCACGAAAGAACTTTCAAAGCTCGAGCTTTGAAAGTTCTTTCGTGGTTTTTTTG-3′;
shAICDA#2, 5′-CCGGCATTTCGTACTTTGGGACTTTCTCGAGAAAGTCCCAAAGTACGAAATGTTTTTG-3′;
shAICDA#3, 5′-CCGGCATTTCGTACTTTGGGACTTTCTCGAGAAAGTCCCAAAGTACGAAATGTTTTTG-3′.

### 2.5. Immunohistochemical Staining and Analysis

All cases of primary and recurrent EGFR-mutant LAC from January 2010 to December 2019 at the Chungnam National University Hospital (Daejeon, South Korea) were reviewed retrospectively. Of these, 42 cases were selected and subjected to immunohistochemical (IHC) analysis. Tissue samples comprised 13 cell blocks and 29 biopsy specimens, and were classified into the following three groups according to clinical and molecular characteristics: Group 1, EGFR-TKI-resistant LACs diagnosed with secondary site T790M EGFR mutation after TKI treatment (*n* = 17) (initially diagnosed with the non-T790M EGFR mutation prior to TKI treatment); Group II, EGFR-TKI-sensitive primary LACs with the non-T790M EGFR mutation (eventually acquiring a secondary T790M mutation during TKI treatment) (*n* = 17); and Group III, primary LACs with a T790M EGFR mutation prior to TKI treatment (*n* = 8). Whole formalin-fixed paraffin-embedded (FFPE) tissues were sectioned, mounted on coated slides, deparaffinized with xylene, hydrated through a graded series of alcohol solutions, and heated for 3 min at full power in a pressure cooker containing 10 mmol/L sodium citrate (pH 6.0) for antigen retrieval. Endogenous peroxidase activity was blocked for 5 min using 0.03% hydrogen peroxide containing sodium azide. The sections were incubated at room temperature for 4 h with antibodies specific to NF-κB p65, NF-κB p105/p50, NF-κB p100/p52, RELB, AICDA, IL-6, p-Jak2, Jak2, p-Stat3, Stat3, and cytidine deaminase (CyD). Detailed information for the antibodies is listed in Supplementary Table S2.

After washing, the samples were incubated with an HRP-labeled polymer (antimouse; Dako EnVision+system-HRP (DAB); Dako, Carpinteria, CA, USA) for an additional 20 min at room temperature, followed by additional washing. After rinsing, the chromogen was developed for 2 min. The slides were then counterstained with Meyer’s hematoxylin, dehydrated, and cover-slipped. All methods and experiments were conducted in accordance with relevant guidelines and regulations. IHC staining was scored to evaluate both the intensity of the staining and the proportion of stained tumor cells per slide. The staining intensity and proportion were analyzed by histo-score (H-score), which is based on four IHC categories: negative (0), weak (1+), moderate (2+), and strong (3+). In each case, an H-score (potential range, 0–300) was calculated as follows: H-score = ((1 × % weakly stained cells) + (2 × % moderately stained cells) + (3 × % strongly stained cells)) [20].

### 2.6. Statistical Analysis

All in vitro experiments were repeated three times, and statistical significance was analyzed using a two-tailed Student’s t-test. A *p*-value <0.05 was considered statistically significant (* *p* < 0.05, ** *p* < 0.01, *** *p* < 0.001). SPSS software (version 26; IBM Corp., Armonk, NY, USA) and GraphPad Prism version 5.0 software (GraphPad, San Diego, CA, USA) were used for all statistical analyses.

## 3. Results

### 3.1. Expression of NF-κB, AICDA, Akt, JAK2/STAT3, and IL6 by EGFR-Mutated LACs

**Figure 1:**
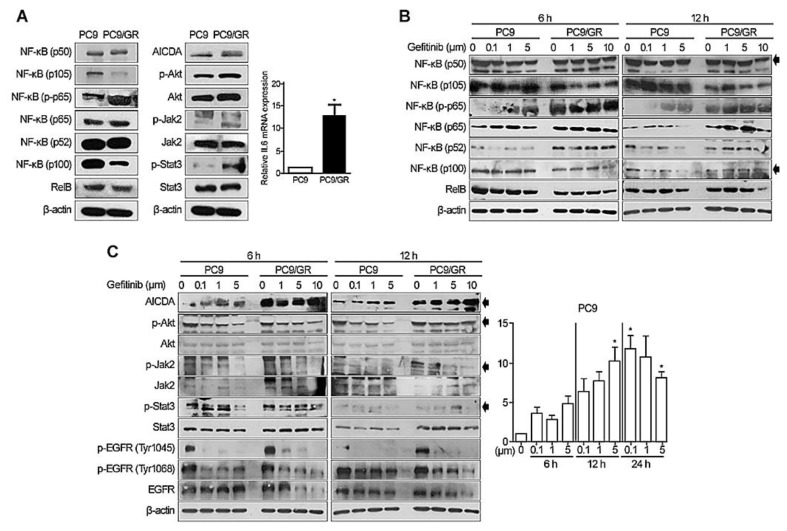
Expression of NF-κB, AICDA, Akt, JAK2/STAT3, and IL6 by lung adenocarcinoma cell lines. (**A**) Basal expression of NF-κB components, AICDA, p-Akt, Akt, p-Jak2, Jak2, p-Stat3, and Stat3, and relative expression of IL-6 mRNA, by the PC9 (adenocarcinoma with an EGFR exon 19 deletion) and PC9/GR (EGFR-TKI-resistant adenocarcinoma with the T790M mutation) cell lines. Changes in expression in (**B**) NF-κB components and (**C**) AICDA, p-Akt, Akt, p-Jak2, Jak2, p-Stat3, and Stat3, and in relative expression of IL-6, after treatment with gefitinib (EGFR-TKI). Uncropped Western blot films are listed in Supplementary Figure S2A–C. β-actin was used as a loading control. Two-tailed Student’s *t*-test was used to determine statistical significance. A *p*-value < 0.05 was considered statistically significant (* *p* < 0.005).

First, we examined the expression of NF-κB components, AICDA, Akt, JAK2/STAT3, and IL6 in PC9 (adenocarcinoma with the EGFR exon 19 deletion) and PC9/GR (adenocarcinoma with the EGFR-TKI-resistant with T790M mutation) cells, using Western blotting and RT-PCR (Figure 1A). PC9/GR cells showed a significantly higher expression of p50, p105, p65, and p-p65 NF-κB proteins, all of which are components of the canonical NF-κB pathway, than PC9 cells. PC9 cells showed a higher expression of p100 NF-κB protein, a component of the noncanonical pathway, than PC9/GR cells. Basal AICDA expression in PC9/GR cells was higher than that in PC9 cells. PC9/GR cells showed a higher expression of p-Akt, Akt, pJak2, and pStat3 than PC9 cells. The expression of IL-6 mRNA was significantly higher in PC9/GR cells.

To evaluate the expression of NF-κB components and other signaling molecules during EGFR-TKI therapy, we examined temporal changes in protein expression in PC9 and PC9/GR cells exposed to an EGFR-TKI (gefitinib) (Figure 1B,C). The expression of canonical p50, p105, and p-p65 NF-κB proteins by PC9 and PC9/GR cells increased in a TKI dose-dependent manner. By contrast, the expression of noncanonical NF-κB (p100 and RELB) proteins fell slightly over time. Also, we found a dose-dependent increase in AICDA expression, and a decrease in p-Akt, p-Jak2, p-Stat3, and Stat3 expression, in PC9 and PC9/GR cells treated with the EGFR-TKI (Figure 1C). The relative expression of IL-6 mRNA increased significantly at 12 h following EGFR-TKI exposure but decreased after 24 h (* *p* < 0.005); this was also dose-dependent. The supplementary data reveal detailed repetitive results of Western blot analysis (Figure S1A,B).

### 3.2. Knockdown of NF-κB and AICDA Restores the Sensitivity of TKI-Resistant Lung Cancer Cells to EGFR-TKI

**Figure 2:**
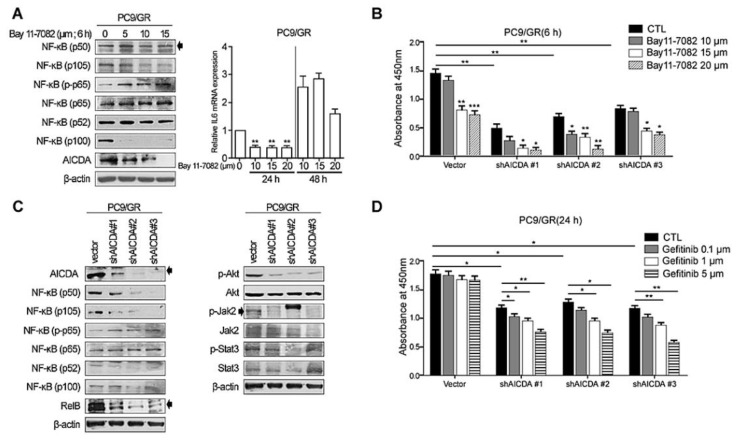
Knockdown of NF-κB and AICDA activity in EGFR-TKI-resistant lung adenocarcinoma cells. (**A**) Treatment of EGFR-TKI-resistant PC9/GR cells with an NF-κB inhibitor (Bay 11-7082), and changes in expression of (**A**) NF-κB components and IL-6. (**B**) Cell viability assay conducted after knockdown of AICDA. (**C**) Knockdown of AICDA and expression of NF-κB components, p-Akt, Akt, p-Jak2, Jak2, p-Stat3, and Stat3. (**D**) Cell viability assay conducted after knockdown of AICDA plus treatment with an EGFR-TKI (gefitinib). All experiments were performed in triplicate (*n* = 3) and detailed repetitive data are listed in Supplementary Figure S1C–F. Uncropped Western blot films are listed in Supplementary Figure S2D,E. β-actin was used as a loading control. Two-tailed Student’s *t*-test was used to determine statistical significance. A *p*-value < 0.05 was considered statistically significant (* *p* < 0.005, ** *p* < 0.001, *** *p* < 0.001).

Next, we treated EGFR-TKI-resistant PC9/GR cells with an NF-κB inhibitor (Bay 11-7082) (Figure 2A). PC9/GR cells showed a decreased expression of canonical (p105) and noncanonical (p100) NF-κB proteins, and of AICDA, in the presence of the NF-κB inhibitor. These effects were dose-dependent (Figure 2A, left panel). The relative expression of IL-6 mRNA decreased significantly and dose-dependently at 24 h following exposure to the NF-κB inhibitor but decreased after 48 h in PC9/GR cells (* *p* < 0.005) (Figure 2A, right panel). The cell viability assay showed a significant decrease in PC9/GR cell viability in the presence of the NF-κB inhibitor (Figure 2B). After treatment with a high concentration of inhibitors, cell proliferation was inhibited, and it was confirmed that many cells fell into apoptosis. Moreover, cell death increased further after knockdown of AICDA (using shAICDA) and treatment with the NF-κB inhibitor (Figure 2B). Because the NF-κB inhibitor decreased AICDA expression, knockdown of AICDA significantly decreased the expression of canonical (p50 and p105) NF-κB proteins in PC9/GR cells (Figure 2C, left panel). In addition, knockdown of AICDA resulted in decreased p-Akt expression (Figure 2C, right panel). The reduced viability of shAICDA-treated cells suggests the restoration of gefitinib sensitivity (Figure 2D). Moreover, the decrease in viability of shAICDA cells was proportional to the dose of gefitinib.

### 3.3. Therapeutic Effects of Combined Treatment with an EGFR-TKI Plus Cosuppression of NF-κB and AICDA

**Figure 3:**
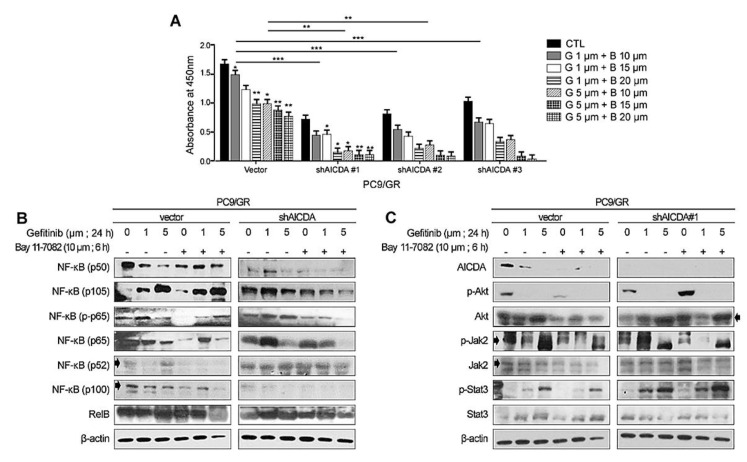
Effect of cosuppression of NF-κB and AICDA plus treatment with an EGFR-TKI on resistant adenocarcinoma cells. (**A**) Cell viability assay conducted after combination therapy with an EGFR-TKI (G, gefitinib) plus NF-κB inhibition (**B**, Bay11-7082) and AICDA knockdown, and (**B**,**C**) its effect on expression of NF-κB components, p-Akt, Akt, p-Jak2, Jak2, p-Stat3, and Stat3. Cell viability assay was performed in triplicate (*n* = 3) and detailed data are listed in Supplementary Figure S1G. Uncropped Western blot films are listed in Supplementary Figure S1H. Two-tailed Student’s *t*-test was used to determine statistical significance. A *p*-value < 0.05 was considered statistically significant (* *p* < 0.005, ** *p* < 0.001, *** *p* < 0.0001).

To evaluate the effect of combination therapy, we examined cell proliferation in the presence of an EGFR-TKI plus suppression of NF-κB and AICDA. PC9/GR cells showed the greatest dose-dependent decrease in cell viability after combination therapy (Figure 3A). Western blot analysis confirmed that canonical NF-κB expression components (p50, p105, p-p65, p65) showed the greatest decrease in expression after treatment with an EGFR-TKI plus NF-κB/AICDA suppression (Figure 3B). The other signaling molecules showed somewhat different patterns of expression under combination treatment. AICDA expression fell after AICDA knockdown and treatment with an NF-κB inhibitor (Figure 3C). The expression of Akt and p-Akt decreased under conditions of EGFR-TKI and NF-κB suppression. Finally, the expression of Jak2 and Stat3 showed no significant change after combination treatment.

### 3.4. Immunohistochemical Analysis of Specimens Showing Initial and Acquired EGFR-TKI Resistance

To validate the clinical relevance of our findings, we examined the expression of NF-κB components and other antibodies in 42 cases of LAC tissue. Among them, a total 33 cases (26 matched cases from 13 patients before and after acquisition of the T790M mutation, and 7 cases with pre-treatment biopsy specimens harboring a primary T790M EGFR mutation) were successfully examined through IHC analysis.

**Figure 4:**
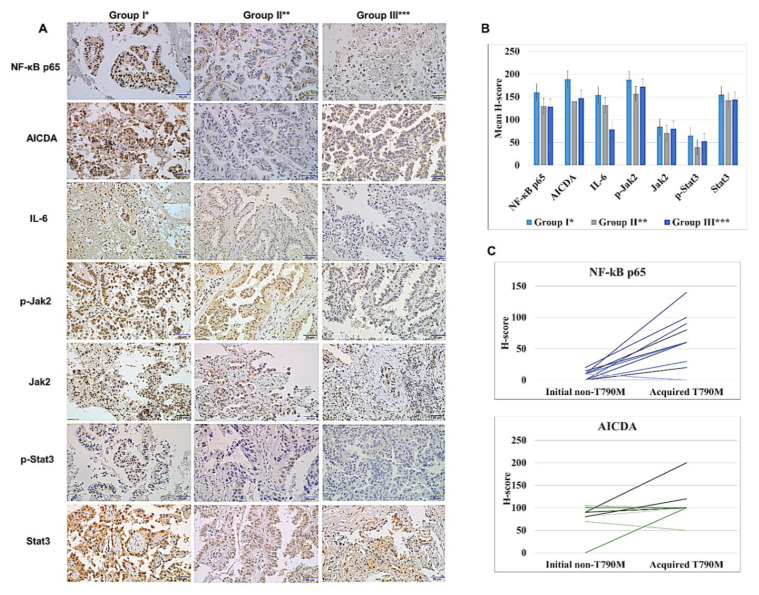
(**A**) Immunohistochemical results of the antibodies by patient groups (×400, scale bar: 50 µm). (**B**) Mean ± standard deviation of the immunologic H-scores by patient groups. (**C**) Matched analysis of NF-κB p65 and AICDA expression at baseline (initial non-T790M) and after acquired EGFR-TKI resistance (*n* = 13). Group I*, EGFR-TKI-resistant LACs diagnosed with secondary site T790M EGFR mutation after TKI treatment; Group II**, EGFR-TKI-sensitive primary LACs with the non-T790M EGFR mutation; and Group III***, primary LACs with a T790M EGFR mutation prior to TKI treatment.

The antibodies including NF-κB p65, AICDA, IL-6, p-Jak2, Jak2, p-Stat3, and Stat3 showed differential immunologic expression by patient groups, and the mean H-scores for Group I (LAC tissues with the acquired T790M EGFR mutation) were higher than that of the other groups (Figure 4A,B). On the other hand, regardless of the type of initial EGFR mutation, the IHC results were similar in Group II and Group III.

Although NF-κB p65 and AICDA tended to increase consistently after acquisition of the T790M mutation (Figure 4C), the trend was rather inconsistent for IL-6, p-Jak2, Jak2, p-Stat3, and Stat3. Other antibodies including NF-κB p105/p50 and NF-κB p100/p52 showed slightly differential immunologic expression by patient groups, but the differences were considered insignificant. The supplementary data reveal details for the IHC results (Table S3 and Figure S3).

## 4. Discussion

Initially, patients with EGFR-mutated NSCLC show a good response to EGFR-TKI therapy; however, the tumors inevitably acquire drug resistance [2,3,4,5]. Attempts to overcome TKI resistance focus on the development of next-generation EGFR-TKIs, which show therapeutic activity against reactivated EGFR-mutated lung cancers; however, treatment results in the acquisition of further resistance through additional EGFR mutations, as well as the activation of EGFR-independent bypass signaling [21].

In this study, we found that EGFR-TKIs enhanced the expression of canonical NF-κB components in TKI-resistant PC9 and PC9/GR LACs. The expression of canonical NF-κB component p-p65 (in cell lines) and NF-κB p65 (in patient tissue) proteins was significantly higher in EGFR-TKI-resistant LACs than in TKI-sensitive LACs. In addition, NF-κB p-p65 expression increased in line with the dose of EGFR-TKI. Therapeutic responses of TKI-resistant LACs to EGFR-TKIs were restored by NF-κB inhibition.

In addition, we found that the expression of the AICDA protein was higher in TKI-resistant LACs than in TKI-sensitive LACs. EGFR-TKI treatment led to increased AICDA expression in PC9 and PC9/GR cells. AICDA expression increased in line with the dose of EGFR-TKIs, and therapeutic responses of PC9/GR cells to EGFR-TKIs were restored upon knockdown of AICDA. In addition, knockdown of AICDA inhibited the expression of NF-κB. Thus, AICDA and the NF-κB pathway appear to play roles in the acquired resistance of LACs to EGFR-TKI treatment.

El Kadi and colleagues reported that noncanonical NF-κB signaling is associated mainly with AICDA activation to achieve EGFR-TKI resistance [12]; however, our results show that expression of not only canonical NF-κB components, but also NF-κB p65, increased after EGFR-TKI treatment. In addition, knockdown of AICDA mostly decreased the expression of canonical NF-κB components in PC/GR cells. Therefore, AICDA and the canonical NF-κB pathway appear to be the main intracellular signals that promote resistance to EGFR-TKIs in LACs.

NF-κB has protumorigenic effects; therefore, studies of NF-κB inhibition focus on inhibiting cancer progression by single-agent or combination therapy with proven chemotherapeutic agents [22]. Commercial NF-κB inhibitors of lung cancer have been undergoing clinical trials, and several have been approved by the United States Food and Drug Administration [22]. Our results suggest that targeting NF-κB and AICDA may be a novel strategy to prevent or delay the emergence of EGFR-TKI resistance. However, the basic question remains: what is the therapeutic mechanism underlying the effect of AICDA in EGFR-TKI-resistant lung cancer? Although the AICDA enzyme appears to contribute to the acquired resistance to EGFR-TKI, knockdown of AICDA does not imply genetic and structural recovery from an irreversible altered secondary site T790M mutation in EGFR. Hence, the therapeutic effects of AICDA inhibition suggest a regulatory role in the tumorigenic pathway rather than deaminase activity only. We found that knockdown of AICDA inhibited the NF-κB (canonical and noncanonical) and Akt pathways in LAC cells. Inhibition of NF-κB repressed the expression of IL-6 and AICDA. These findings suggest that AICDA plays diverse roles in LACs during EGFR-TKI treatment.

To identify pathways relevant to AICDA/NF-κB signaling, we evaluated the association between Akt, Jak2, Stat3, and IL-6 expression with that of AICDA and NF-κB. The expression of phospho-Akt in PC9 and PC9/GR cells fell in line with the dose of EGFR-TKI. Furthermore, knockdown of AICDA and NF-κB signaling significantly inhibited p-Akt expression in PC9/GR cells. These findings imply an interaction between Akt and the NF-κB/AICDA pathways in LACs during TKI treatment. The expression of Jak2 and Stat3 also decreased in line with the dose of EGFR-TKI. Because knockdown of AICDA and NF-κB did not affect the expression of Jak2 and Stat3, the JAK2/STAT3 pathway does not appear to be involved in activating the NF-κB/AICDA pathway in LACs during TKI treatment. However, we obtained intriguing data regarding the expression of IL-6 mRNA under treatment with EGFR-TKI plus an NF-κB inhibitor. EGFR-TKI increased the expression of IL-6 mRNA in TKI-sensitive LACs in a dose-dependent manner, peaking at 24 h. By contrast, an NF-κB inhibitor decreased IL-6 mRNA levels in TKI-resistant lung cancer up until 24 h, which then increased again by 48 h. These results may reflect complex signal fluctuations in vivo, as well as a lack of data supporting the activation of Jak2 and Stat3 by IL-6. Nonetheless, activated NF-κB appears to affect IL-6 expression; indeed, our IHC studies showed an increased expression of IL-6, p-Jak2, Jak2, pStat3, and Stat3 in EGFR-TKI-resistant LCAs. Further studies are needed to examine the role of the NF-κB/IL-6/JAK2/STAT3 signaling pathway in LAC treated with EGFR-TKIs.

In addition, we observed the dynamic expression of cytidine deaminase (CDA) in LCAs (Table S3, Figures S3 and S4). The AICDA gene encodes an RNA-editing deaminase that belongs to the CDA family. We evaluated the expression of AICDA and CDA and expected similar results. Although CDA was inhibited by knockdown of AICDA, our results did not support our hypothesis that similar results would be obtained for AICDA. CDA expression was higher in EGFR-mutated LACs than in EGFR-TKI-resistant LACs, but was not affected by EGFR-TKIs. Inhibition of NF-κB decreased the CDA expression in both TKI-sensitive and TKI-resistant LACs, but increased abruptly after 48 h (Figure S4). Although previous studies considered CDA to be a potential marker of chemotherapy resistance in cancers [23], we found that AICDA and CDA functioned differently during TKI treatment of LACs. The underlying mechanism needs to be verified.

## 5. Conclusions

We examined the interrelationship between NF-κB and related pathways such as AICDA, Akt, and IL-6/JAK2/STAT3, and investigated their contributions to the acquisition of EGFR-TKI resistance. The data suggest that complex interrelated pathways participate in the acquisition of EGFR-TKI resistance, and that modulating AICDA and NF-κB signaling may overcome limitations associated with treatment of EGFR-TKI-resistant lung cancers.

## Data Availability

All data are available in the main text or Supplementary Materials. All other data supporting the findings of this study are available from the corresponding author upon reasonable request.

## Supplementary Materials

The following supporting information can be downloaded at: https://www.mdpi.com/article/10.3390/cancers14122940/s1, Figure S1: Detailed repetitive results of Western blot analysis and cell viability assay, Figure S2: Uncropped Western blot images used in this study, Figure S3: Immunologic results by patient groups, Figure S4: Western blot analysis of CDA, Table S1: Detailed primer information used in this study, Table S2: Lists of antibodies used for IHC studies, Table S3: Detailed immunologic H-scores by patients groups.
