# Supplementary material for: Cosuppression of NF-κB and AICDA Overcomes Acquired EGFR-TKI Resistance in Non-Small Cell Lung Cancer
Source: Cancers (Basel). 2022 Jun 14;14(12):2940. doi: 10.3390/cancers14122940 (PMC9221089; doi:10.3390/cancers14122940)

Supplementary figure S1

Figure S1A. Basal expression of the antibodies by repetitive western blotting in PC9 and PC9GR cells

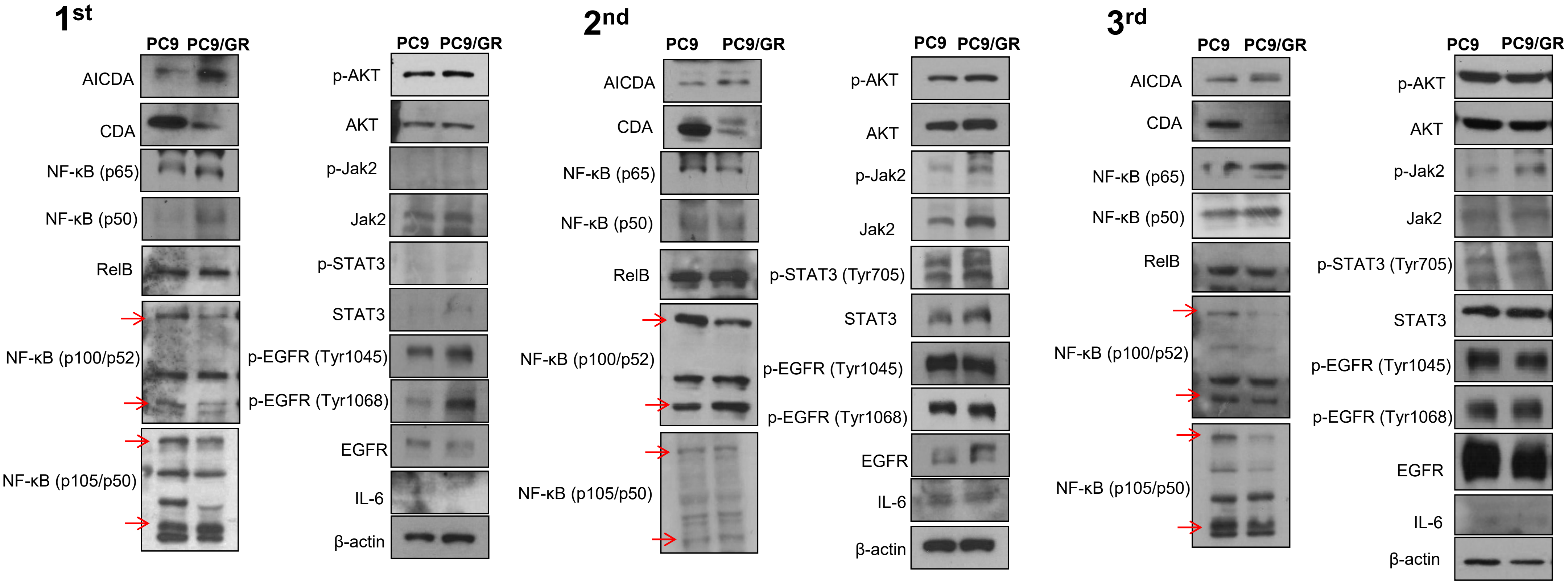

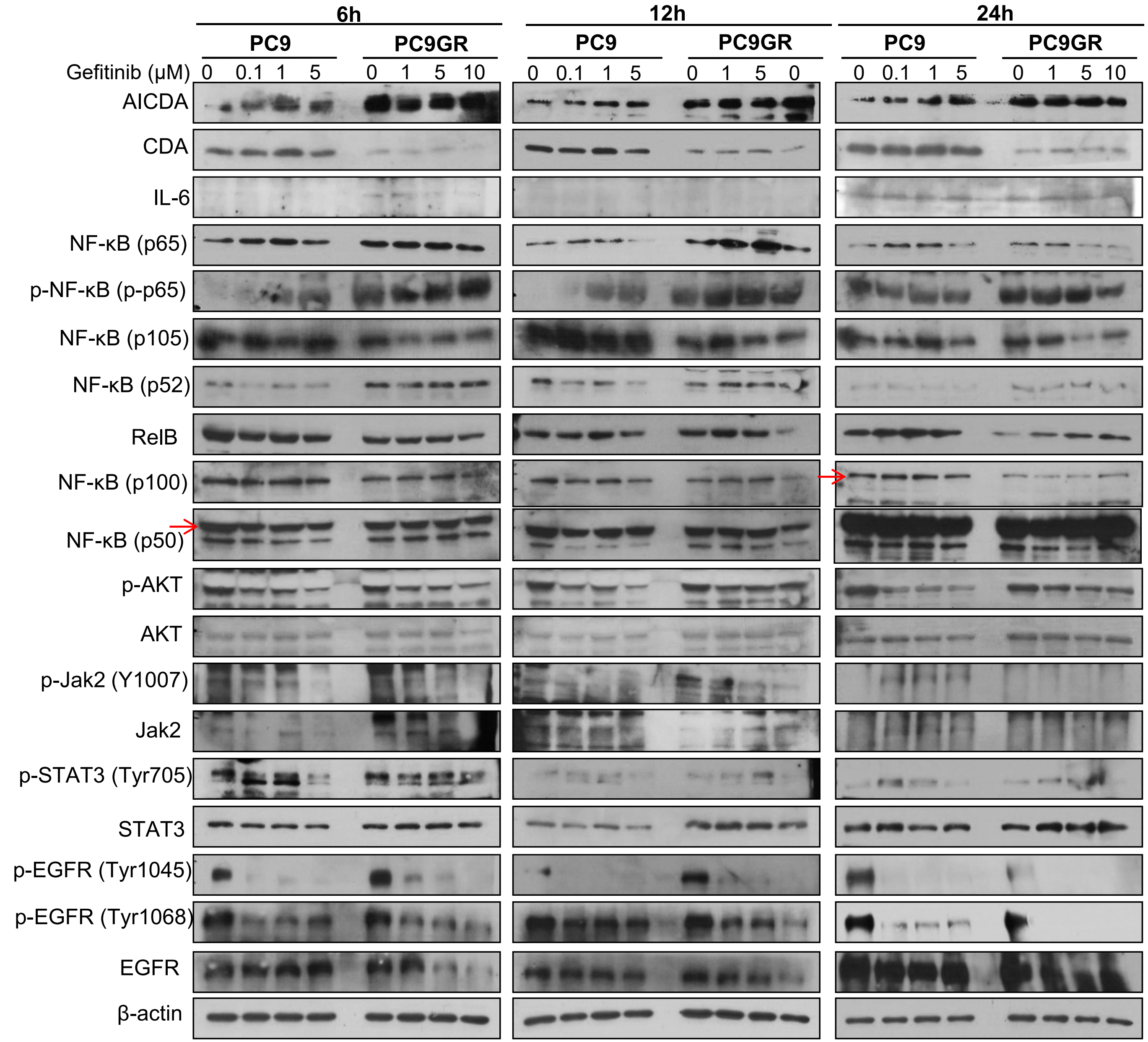

Figure S1B. Changes in expression of the antibodies with Gefitinib in PC9 and PC9GR cells

Figure S1C. Changes in expression of the antibodies with repetitive NF-κB inhibitor in PC9GR cells

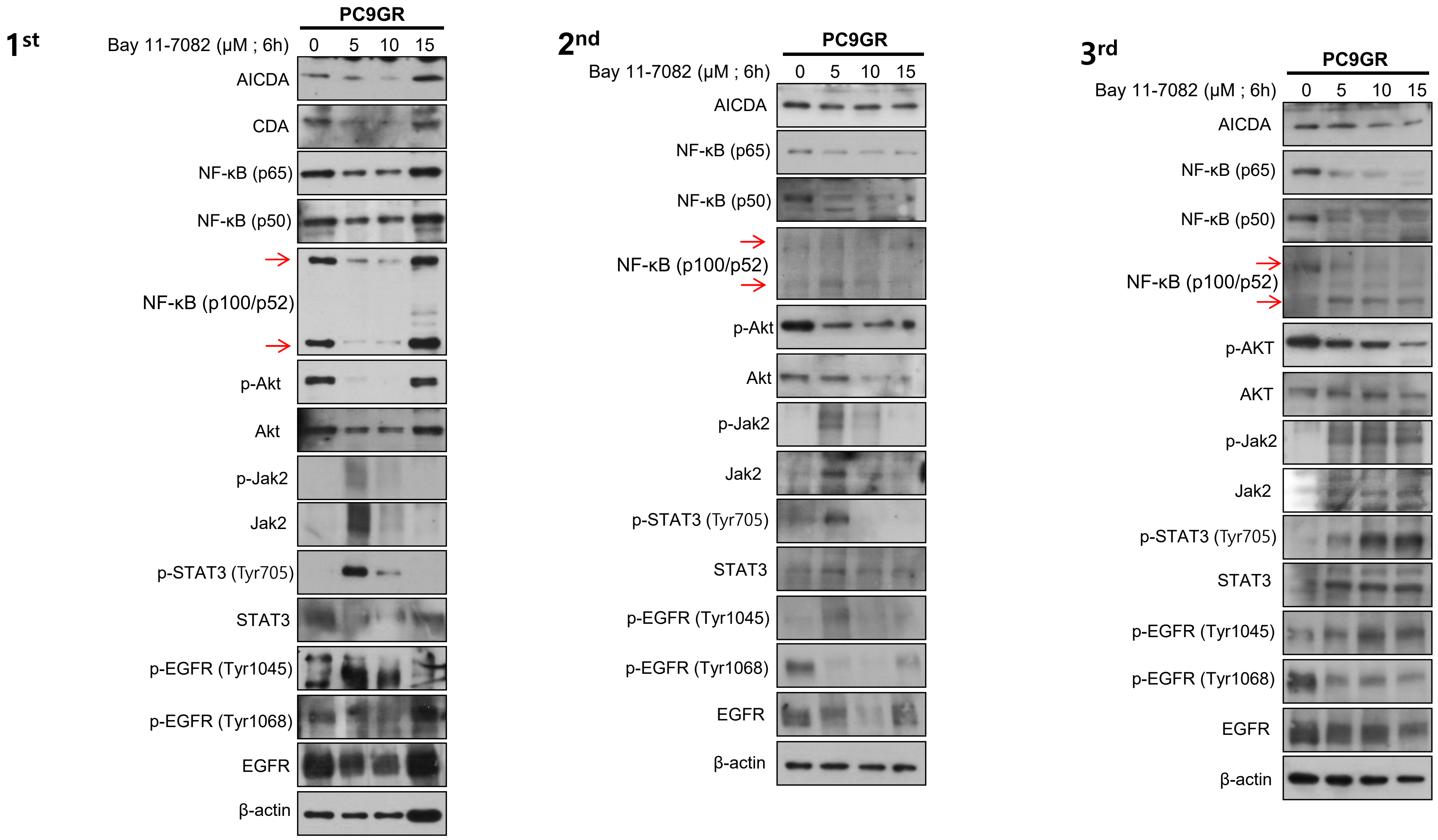

Figure S1C. Changes in expression of the antibodies with repetitive NF-κB inhibitor in PC9GR cells (continued)

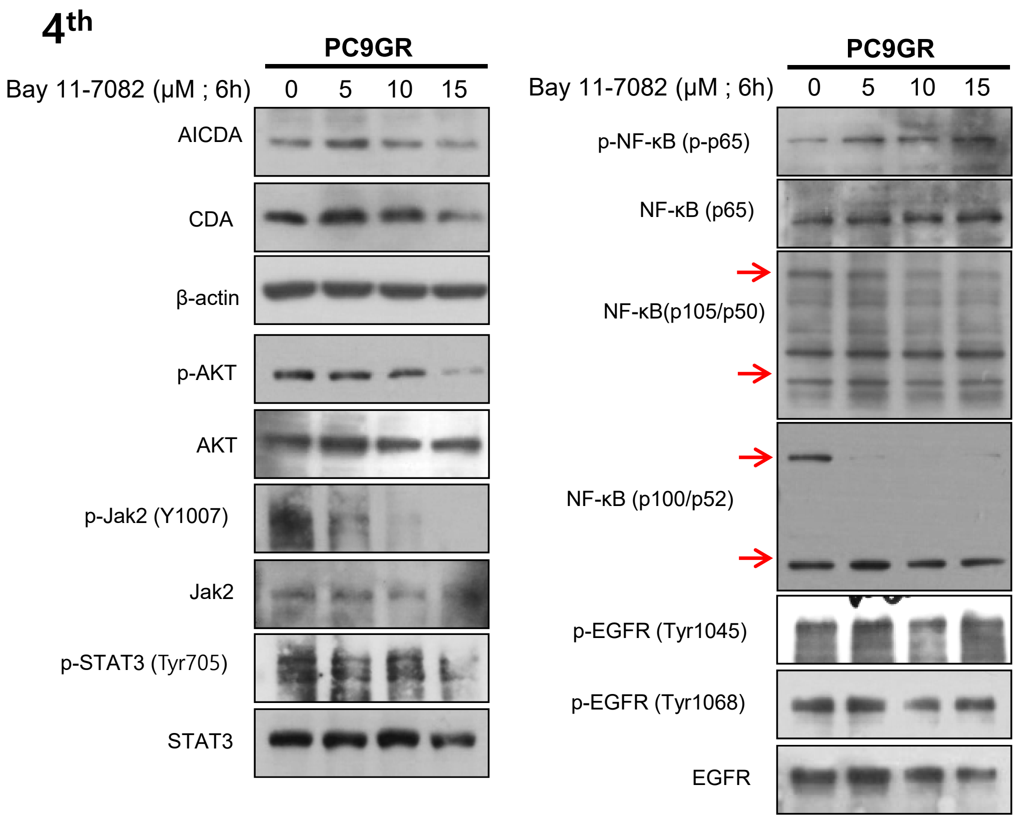

Figure S1D. Repetitive cell viability assay with NF-κB inhibition and AICDA knockdown in PC9GR cells

1st

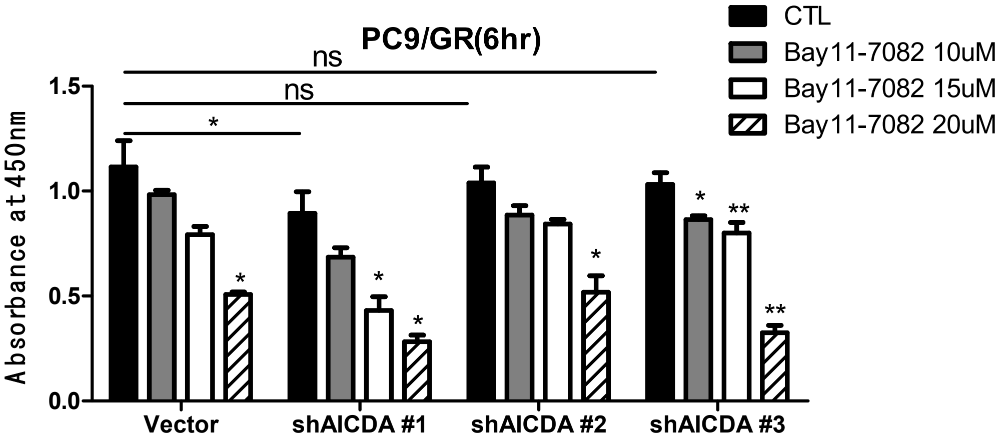

2nd

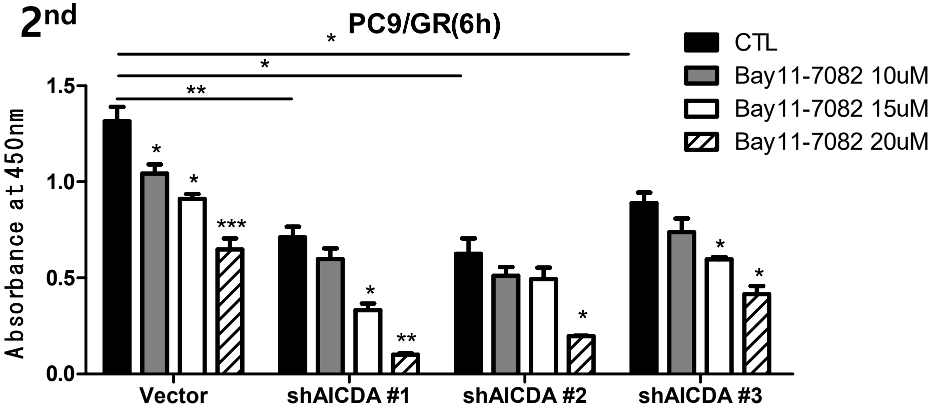

3rd

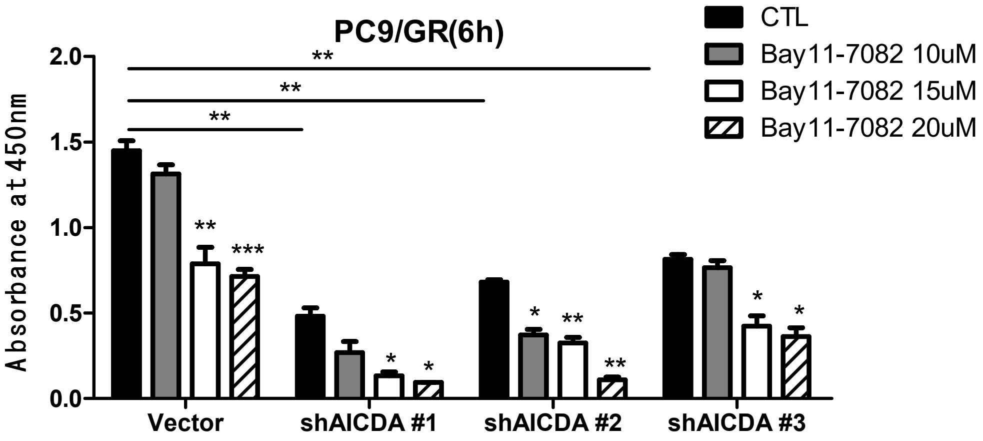

Figure S1E. Changes in expression of the antibodies with repetitive AICDA knockdown in PC9GR cells

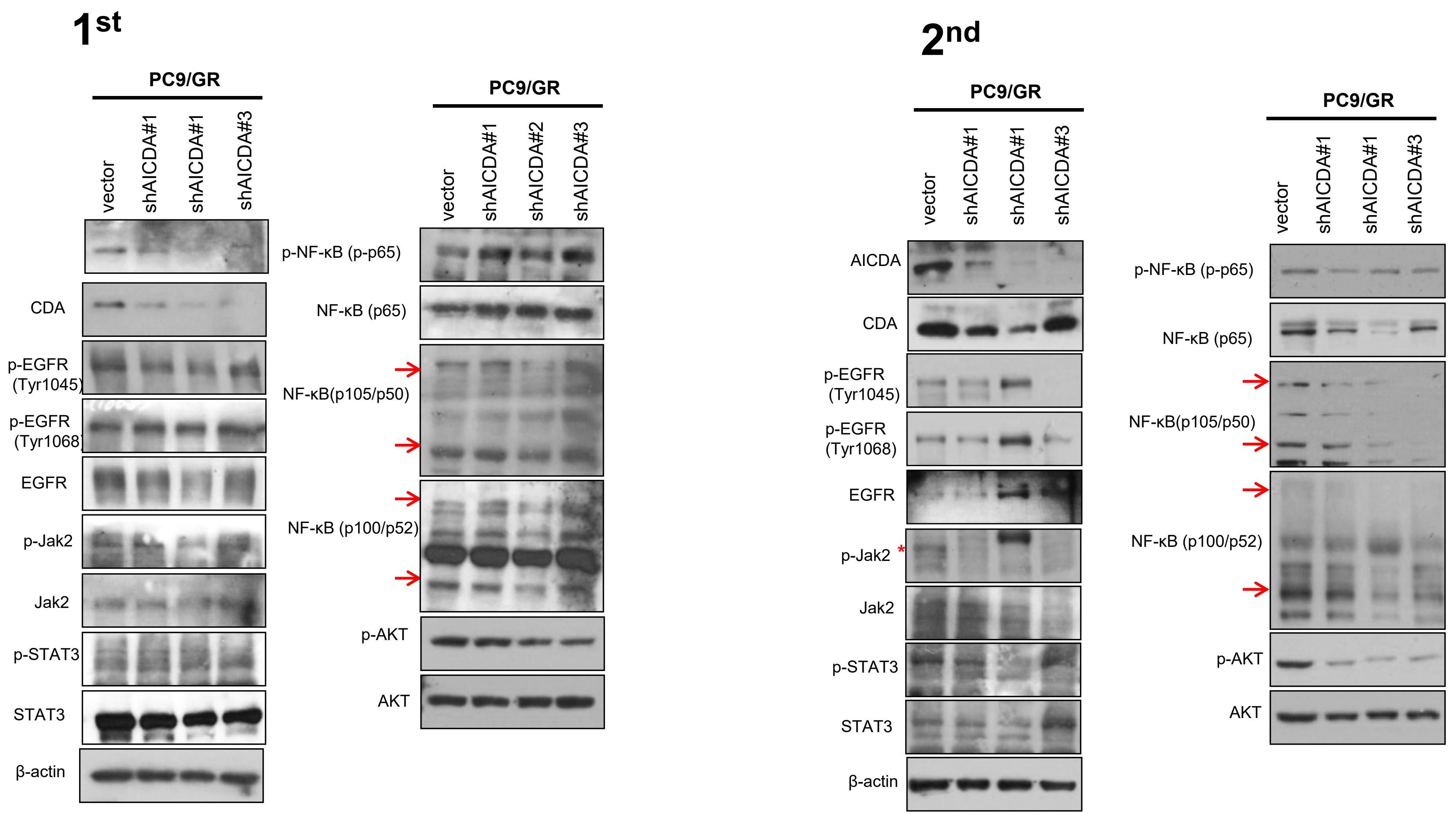

Figure S1E. Changes in expression of the antibodies with repetitive AICDA knockdown in PC9GR cells (continued)

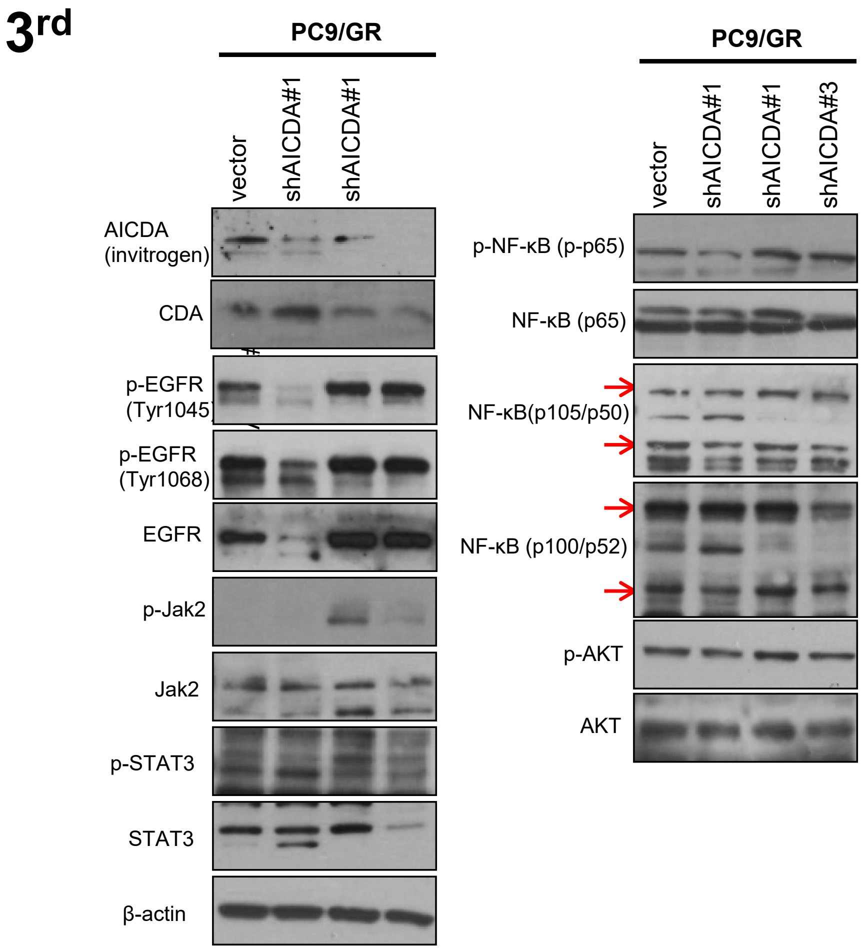

Figure S1F. Repetitive cell viability assay with EGFR inhibition (Gefitinib) and AICDA knockdown in PC9GR cells

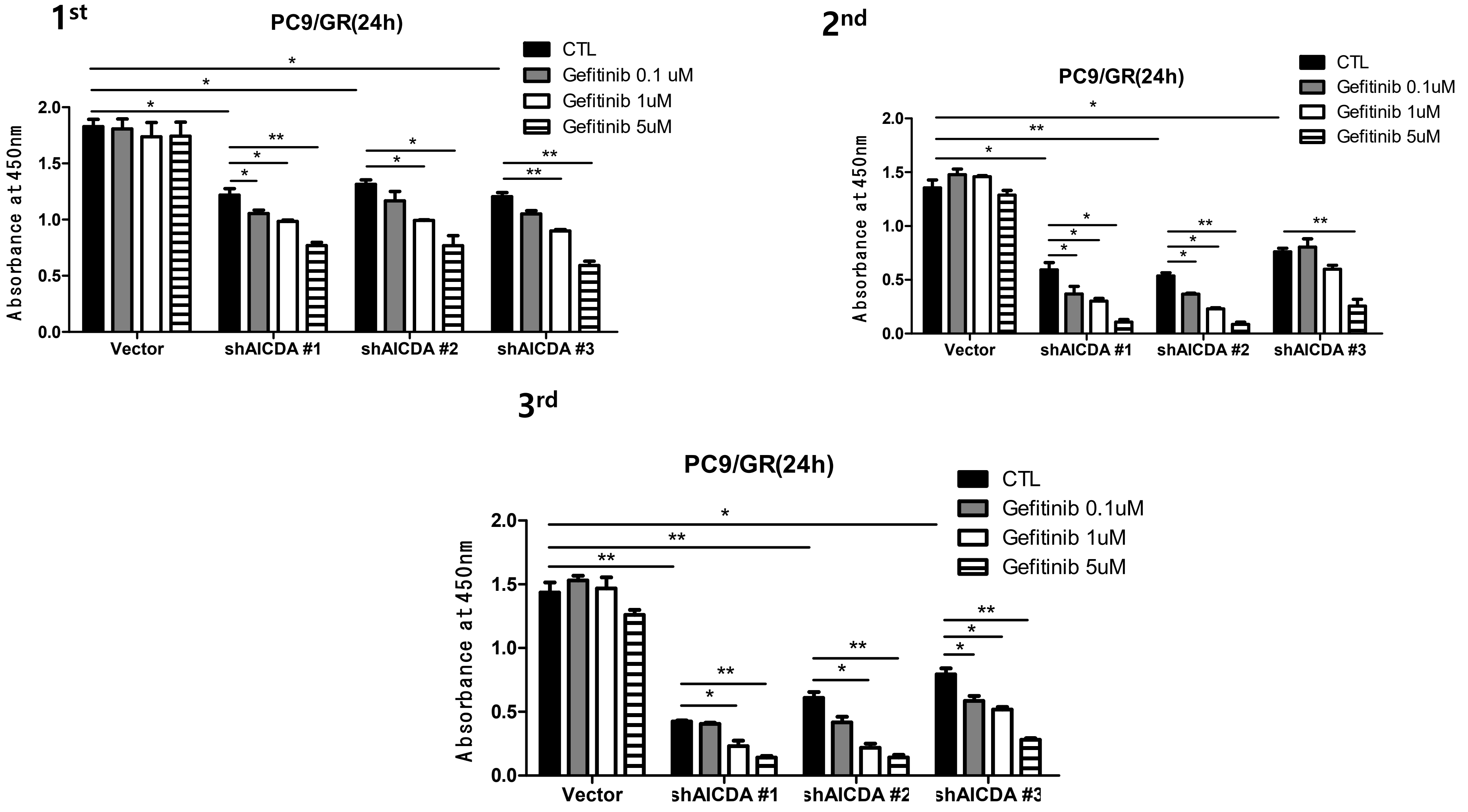

Figure S1G. Repetitive cell viability assay with EGFR inhibition (G), NF-κB inhibition(B) and AICDA knockdown in PC9GR cells

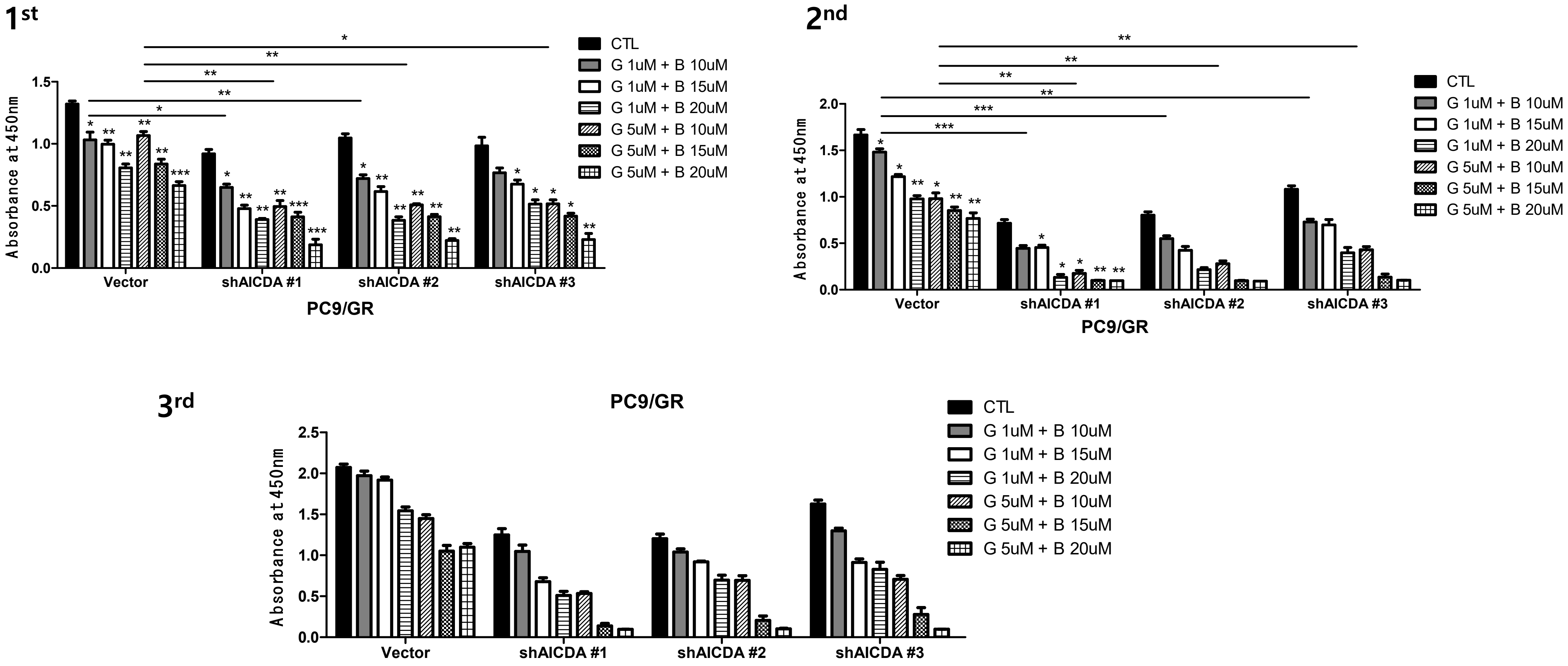

Figure S1H. Changes in expression of the antibodies with EGFR inhibition (Gefitinib), NF-κB inhibition and AICDA knockdown in PC9GR cells

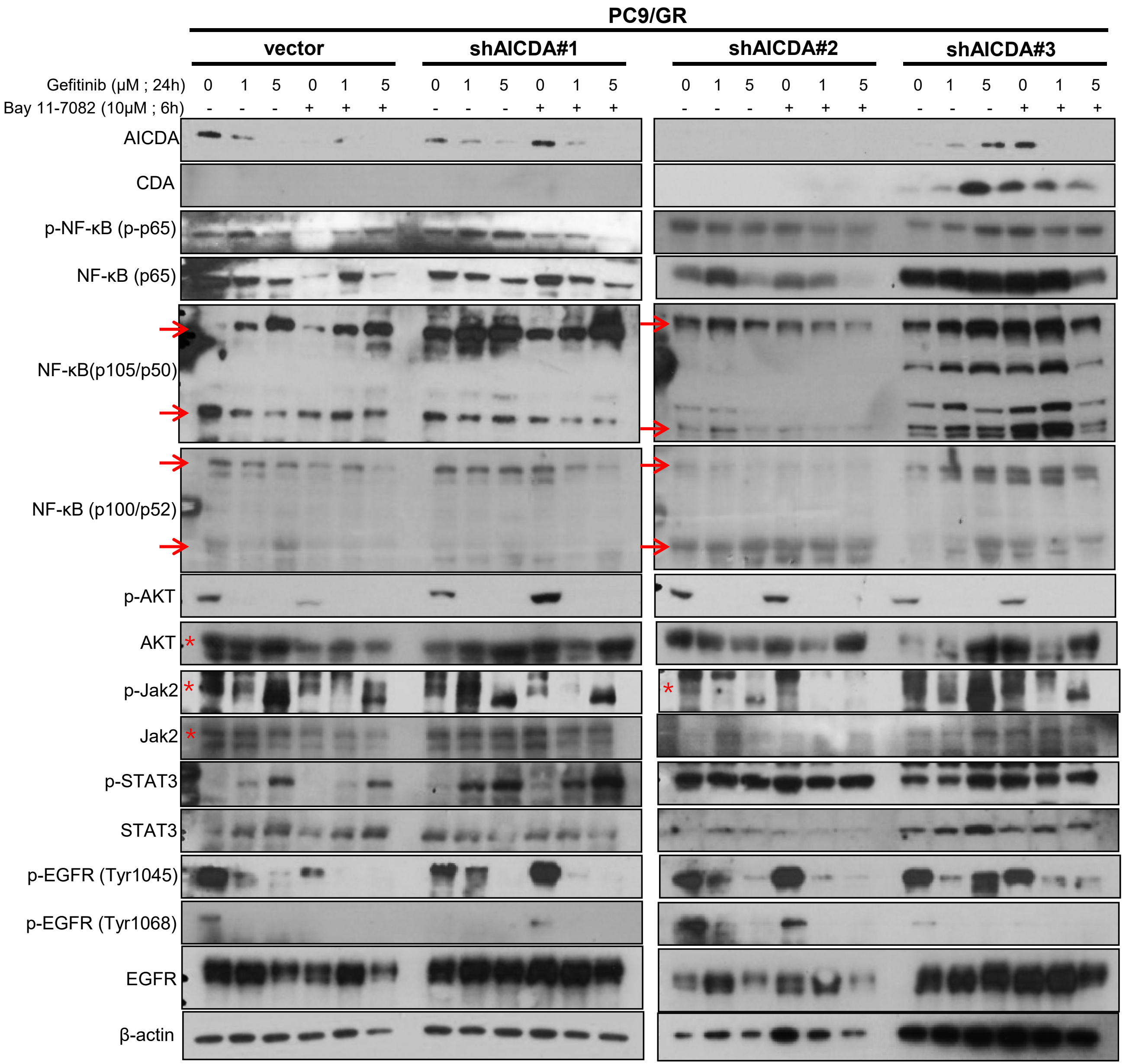

Supplement: Supplementary file 1 [file cancers-14-02940-s001.zip › Figure S1.pdf]
