# Supplementary material for: Cosuppression of NF-κB and AICDA Overcomes Acquired EGFR-TKI Resistance in Non-Small Cell Lung Cancer
Source: Cancers (Basel). 2022 Jun 14;14(12):2940. doi: 10.3390/cancers14122940 (PMC9221089; doi:10.3390/cancers14122940)

**Figure S2A.** Uncropped western blot films for Figure1A

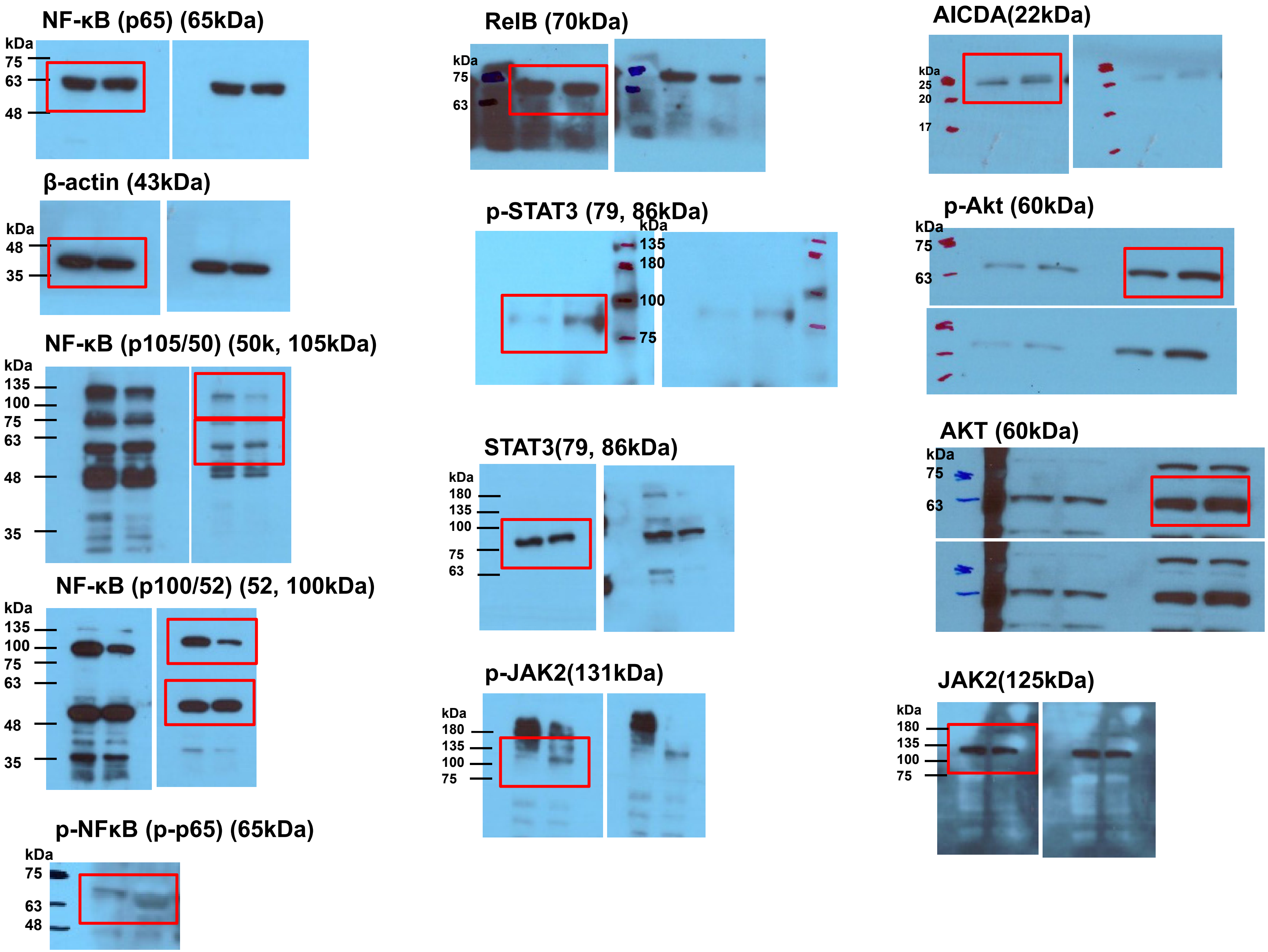

**Figure S2B.** Uncropped western blot films for Figure1B

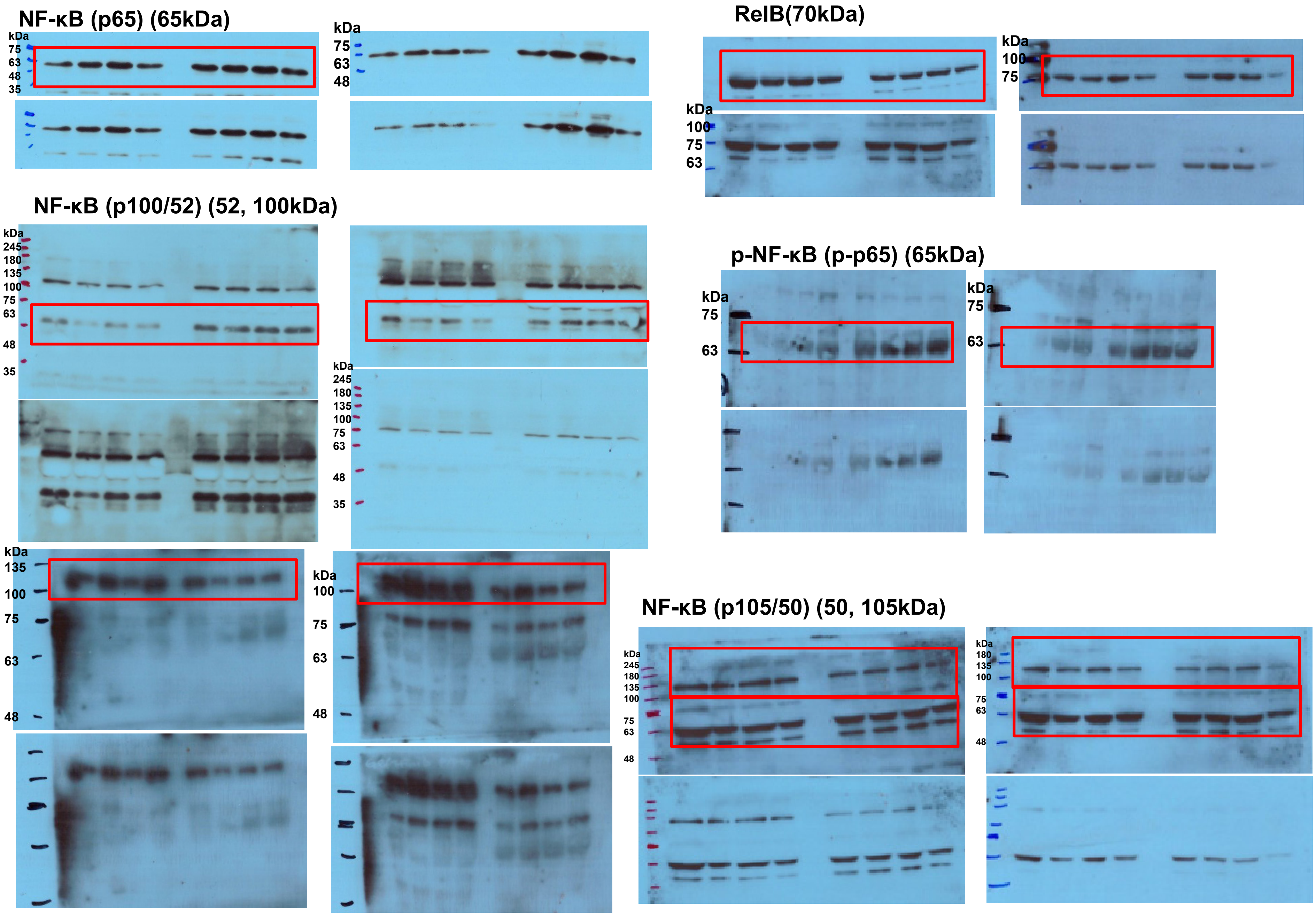

**Figure S2C.** Uncropped western blot films for Figure1C

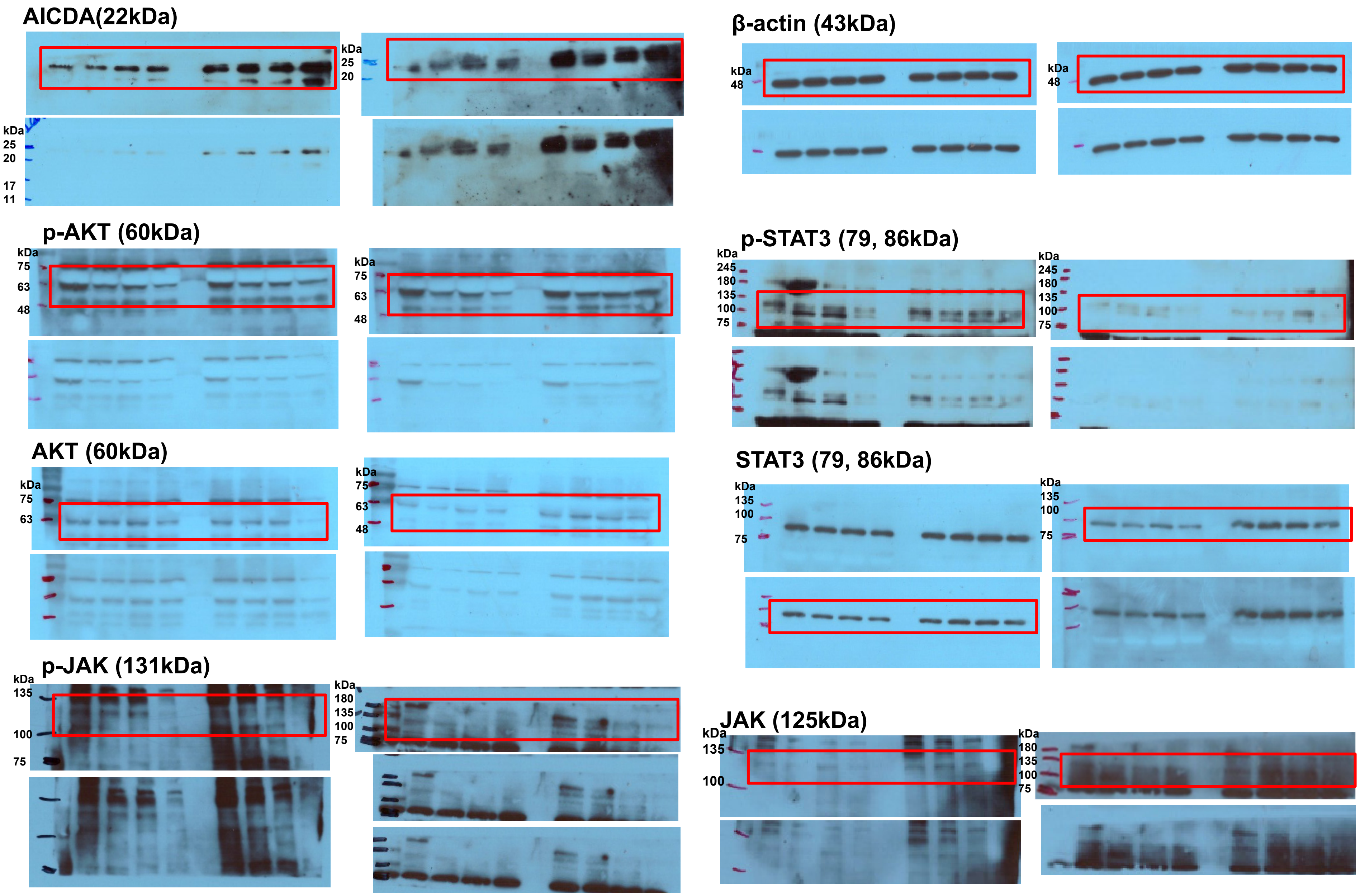

# **Figure S2D.** Uncropped western blot films for Figure2A

**p-NFκB (p-p65) (65kDa)**

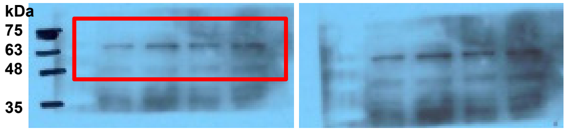


**AICDA(22kDa)**

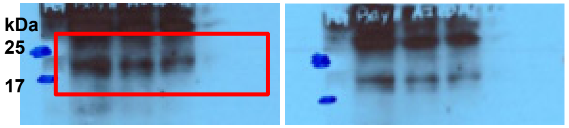


**NF-κB (p65) (65kDa)**

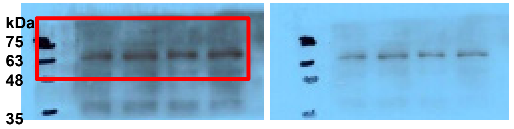


**β-actin (43kDa)**

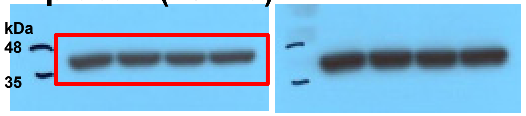


**NF-κB (p105/50) (50,105kDa)**

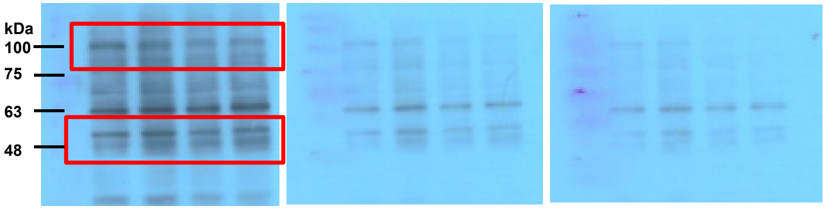


**NF-κB (p100/52) (52,100kDa)**

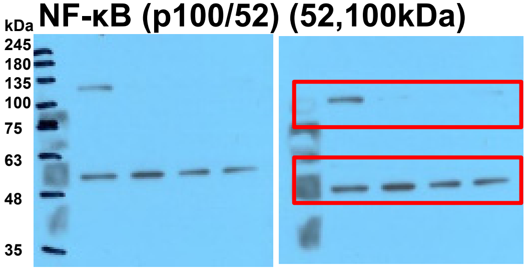

**Figure S2E.** Uncropped western blot films for Figure2C

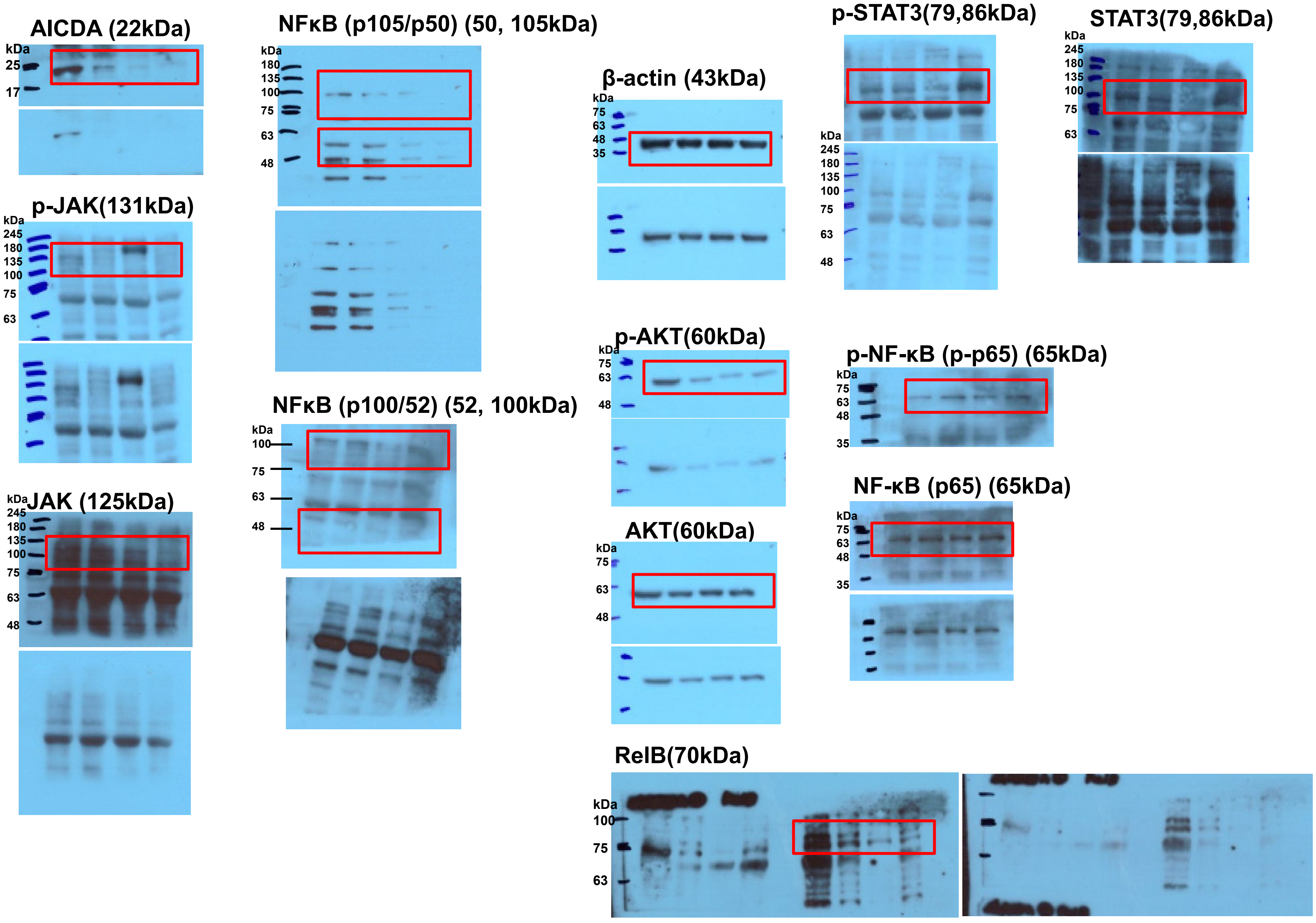

**Figure S2F.** Uncropped western blot films for Figure3B

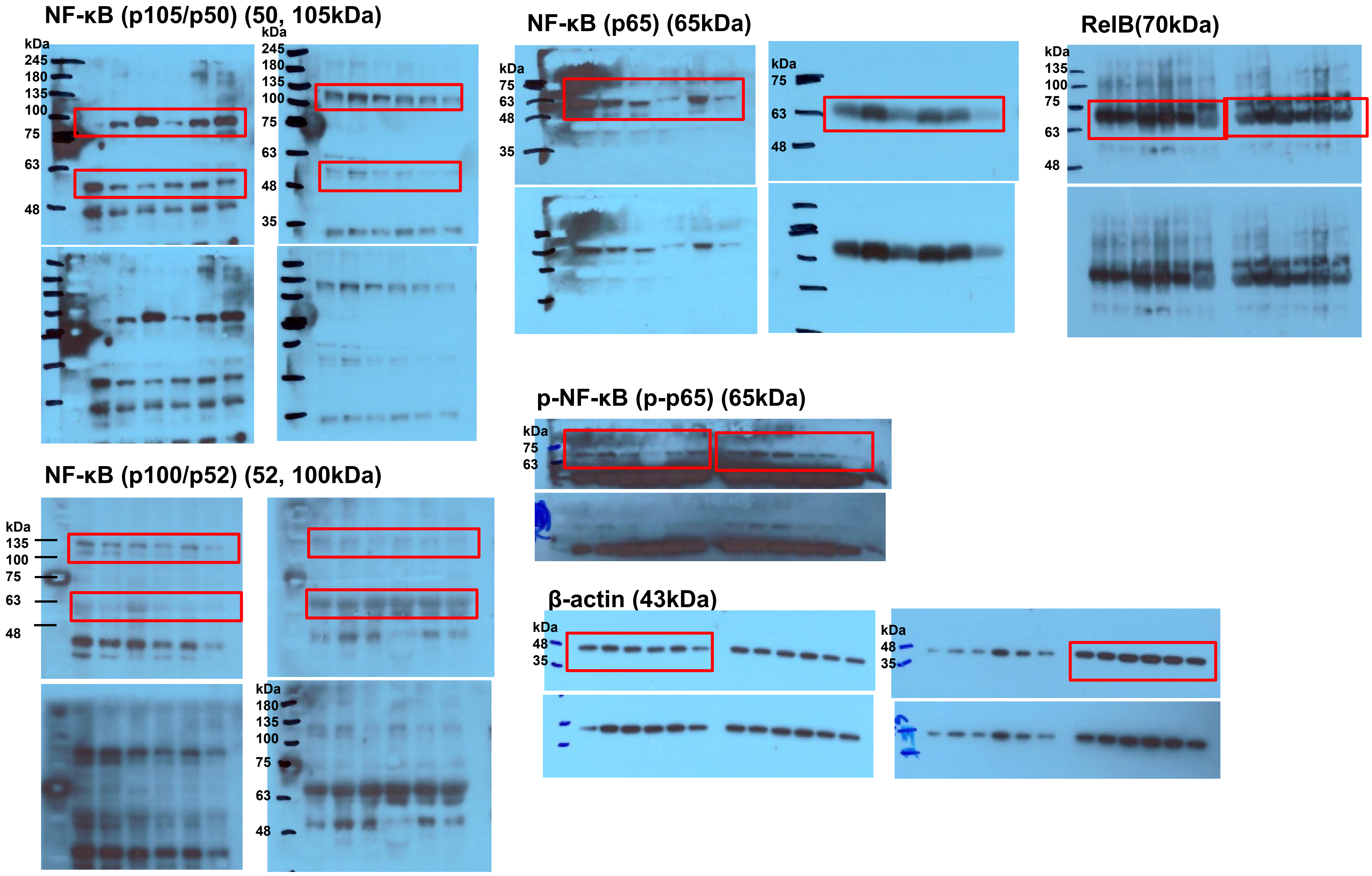

**Figure S2G.** Uncropped western blot films for Figure 3C

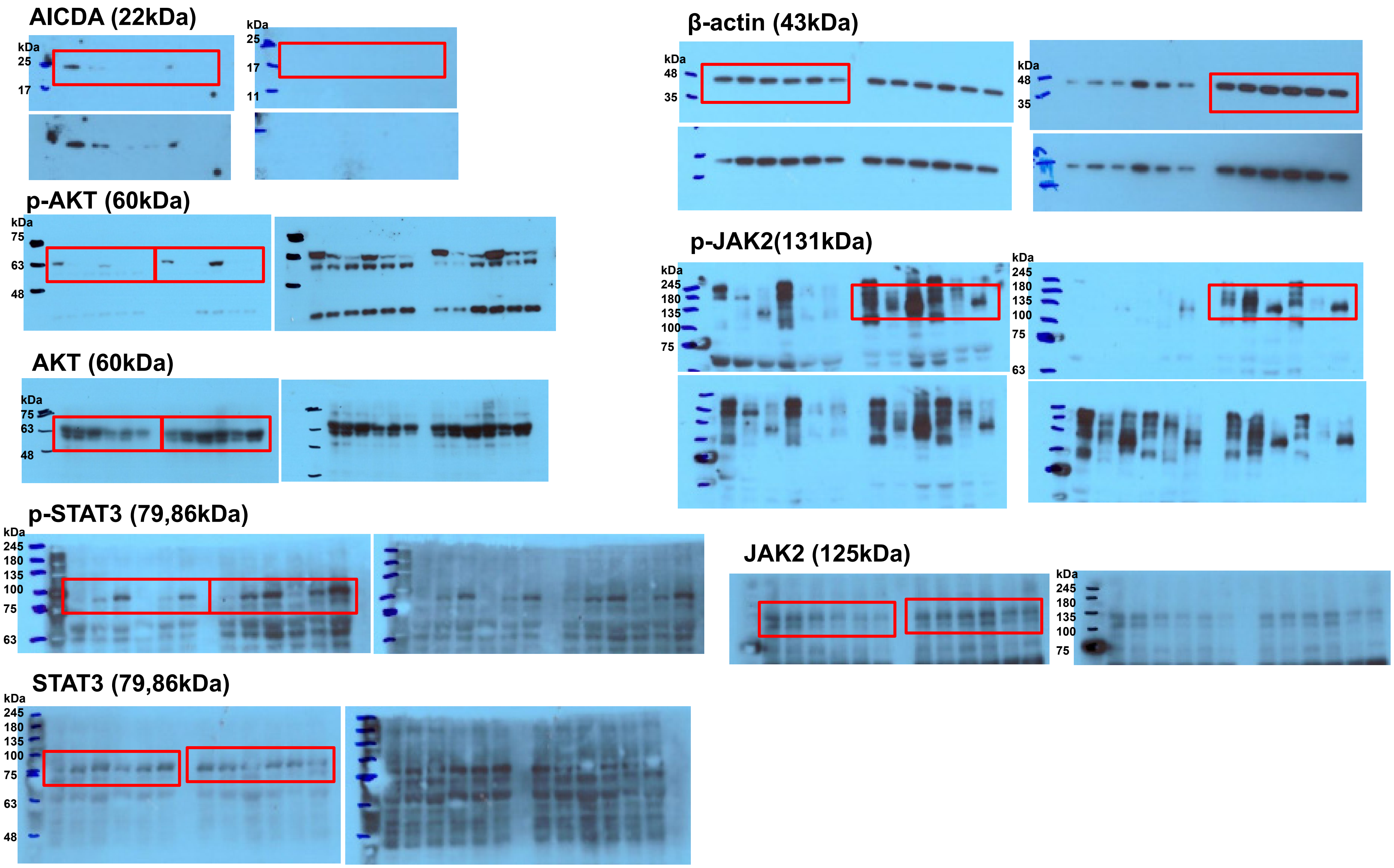

Supplement: Supplementary file 1 [file cancers-14-02940-s001.zip › Figure S2.pdf]
