# Supplementary material for: Cosuppression of NF-κB and AICDA Overcomes Acquired EGFR-TKI Resistance in Non-Small Cell Lung Cancer
Source: Cancers (Basel). 2022 Jun 14;14(12):2940. doi: 10.3390/cancers14122940 (PMC9221089; doi:10.3390/cancers14122940)
Supplement: Supplementary file 1 [file cancers-14-02940-s001.zip › Table S1 and Table S2.pdf]

Supplementary tables

Table S1. Detailed information for primers

Gene	Primer sequence (5'-3')	Size (bp)	NCBI reference
Interleukin-6	5'-AGACAGCCACTCACCTCTTCA-3'	132	NM_000600.5
Interleukin-6	IL6 reverse, 5'-TTC TGC CAG TGC CTC TTT GCT G-3'	132	NM_000600.5
β-actin	forward, 5'-AGG CCC AGA GCA AGA GAG G-3'	229	NM_001101.5
β-actin	reverse, 5'-TAC ATG GCT GGG GTG TTG AA–3'	229	NM_001101.5

Table S2. Detailed information for antibodies

Antibody	Source	Clonality	Dilution	Company	Catalog number
NFκB p65	Rabbit	monoclonal	1:800	Cell signaling technology	#8242
NFκB p105/P50	Rabbit	polyclonal	1:400	Abcam	ab7549
NFκB p100/52	Rabbit	polyclonal	1:100	Cell signaling technology	#4882
RELB	Rabbit	polyclonal	1:1600	Cell signaling technology	#10544
AICDA	Mouse	monoclonal	1:200	Invitrogen	ZA001
IL-6	Rabbit	polyclonal	1:50	Abcam	ab6672
p-Jak2	Rabbit	polyclonal	1:100	Abcam	195055
Jak2	Rabbit	monoclonal	1:200	Cell signaling technology	#3230
p-Stat3	Rabbit	monoclonal	1:200	Cell signaling technology	#9145
Stat3	Rabbit	monoclonal	1:400	Cell signaling technology	#30835
Cytidine deaminase	Rabbit	polyclonal	1:200	Abcam	ab78231

S3 Detailed H-scores by patient groups

Group	NFκB p105/P50	NFκB p65	NFκB p52/p100	RELB	p-STAT3	STAT3	p-jak2	Jak2	il-6	AICDA	CDA
initial non-T790M EGFR mutation (n=13)	165	120	10	0	20	210	215	100	180	150	30
	190	10	0	0	45	200	140	130	210	130	0
	180	180	0	5	0	0	160	100	65	160	0
	170	80	0	0	20	100	210	70	120	200	0
	210	110	10	0	20	170	230	100	110	205	15
	155	180	0	0	50	105	170	90	180	10	50
	140	190	0	0	5	160	120	30	130	130	60
	145	90	0	10	15	40	200	60	105	120	20
	200	110	0	0	0	220	130	80	0	100	0
	190	130	0	30	80	210	70	30	170	110	0
	100	150	0	30	120	180	90	0	140	220	0
	170	100	0	20	50	160	150	60	50	160	150
	200	240	20	0	90	100	150	70	180	130	0
initial T790M EGFR mutation (n=7)	190	90	0	0	10	100	230	100	210	200	100
	185	160	0	10	180	170	200	70	200	130	0
	190	130	5	0	10	140	120	90	100	150	160
	200	210	0	10	30	210	200	100	40	200	30
	110	130	0	0	60	130	230	40	0	130	15
	140	30	0	0	25	170	140	70	0	20	0
	205	150	10	10	55	230	90	90	0	200	0
acquired T790M EGFR mutation (n=13)	100	120	0	0	60	170	130	130	130	205	9
	150	20	0	0	20	100	190	160	80	200	15
	100	140	0	0	10	210	190	100	210	120	90
	205	300	0	0	85	250	215	0	130	210	0
	120	220	0	5	150	210	95	0	110	200	60
	120	160	20	0	0	30	215	150	120	200	0
	200	300	0	0	30	300	220	130	150	200	0
	130	220	10	0	80	270	210	100	170	120	10
	200	90	0	0	90	140	220	150	200	200	130
	180	200	20	0	110	130	220	70	190	200	15
	170	200	0	0	130	140	210	80	165	200	0
	205	10	0	10	70	0	190	30	180	200	0
	180	100	0	0	10	70	135	0	170	200	0
