# Supplementary figures and images for: Cosuppression of NF-κB and AICDA Overcomes Acquired EGFR-TKI Resistance in Non-Small Cell Lung Cancer
Source: Cancers (Basel). 2022 Jun 14;14(12):2940. doi: 10.3390/cancers14122940 (PMC9221089; doi:10.3390/cancers14122940)

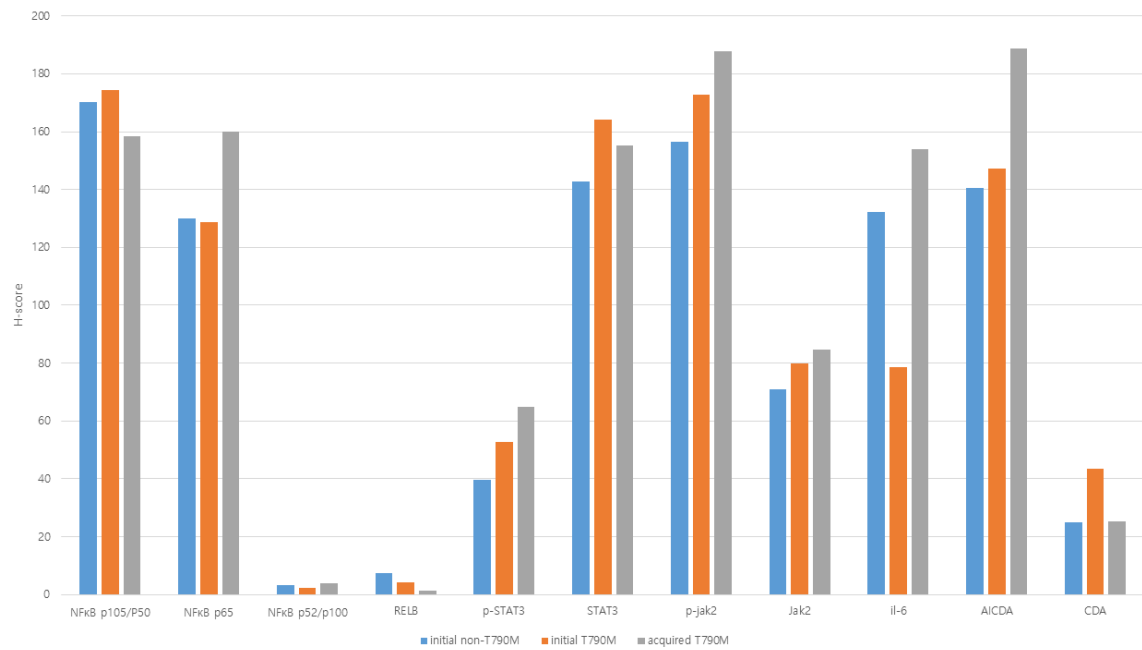

**Figure S3.** Immunologic results by patient groups.

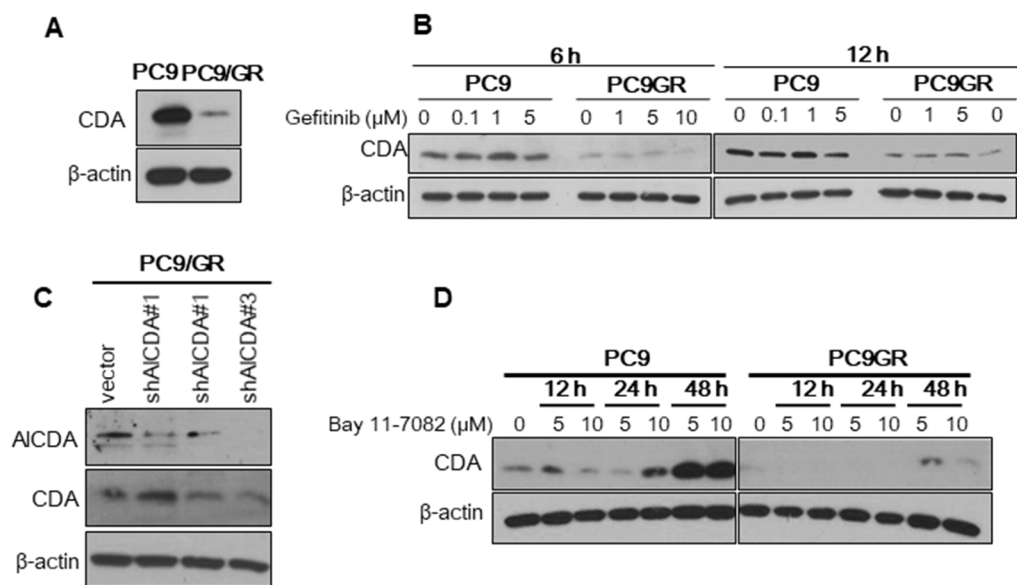

**Figure S4.** Western blot analysis of CDA.

Supplement: Supplementary file 1 [file cancers-14-02940-s001.zip › Figure S3 and Figure S4.pdf]
